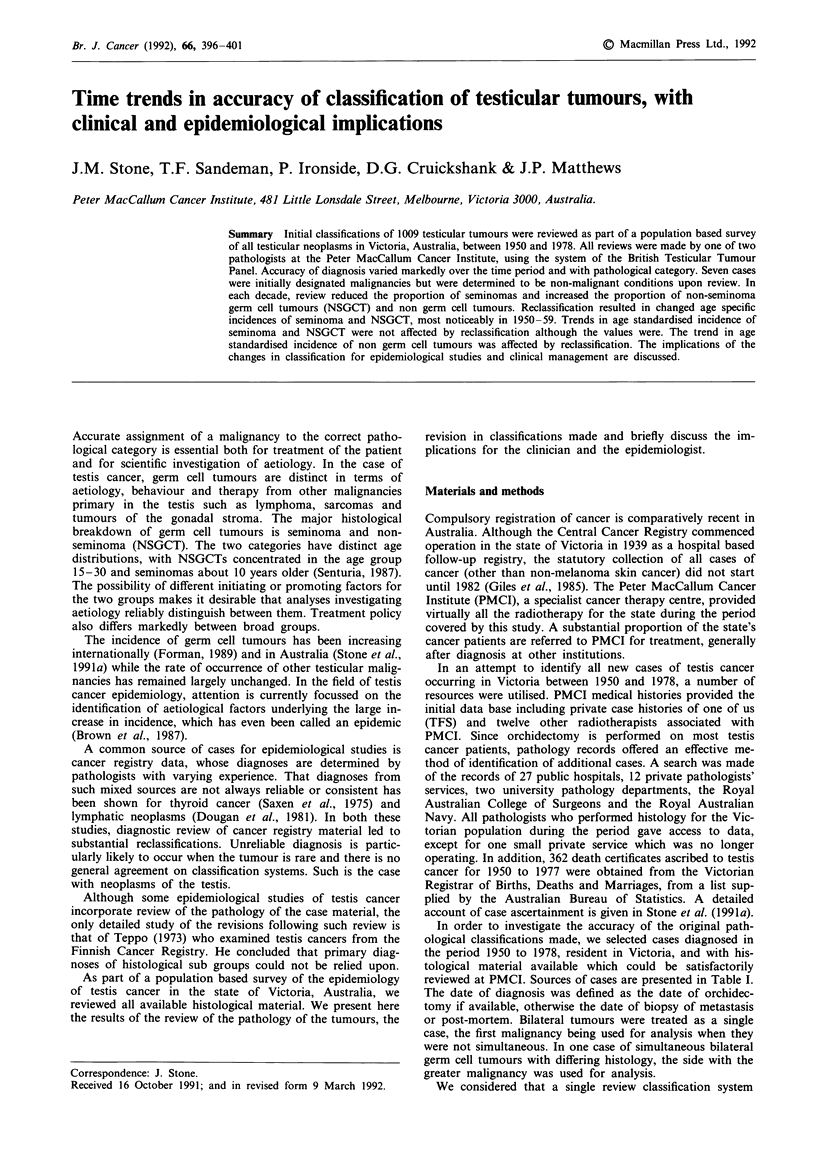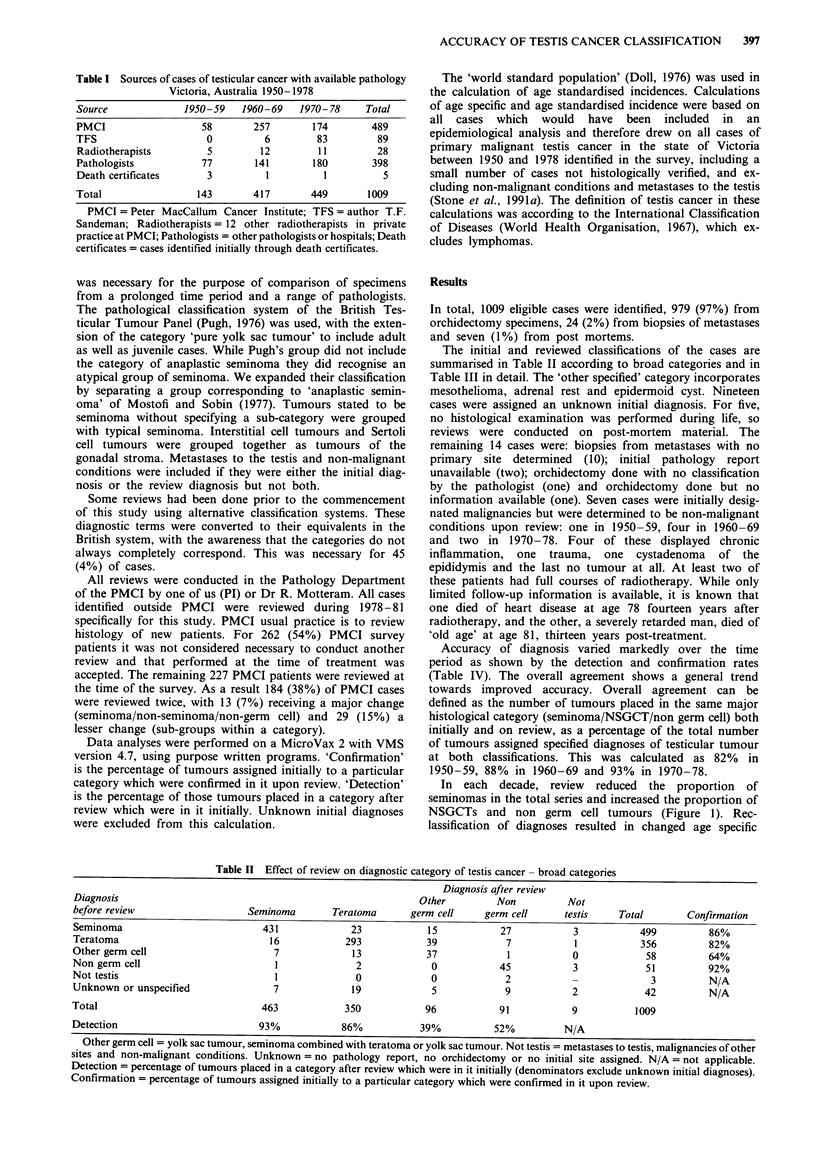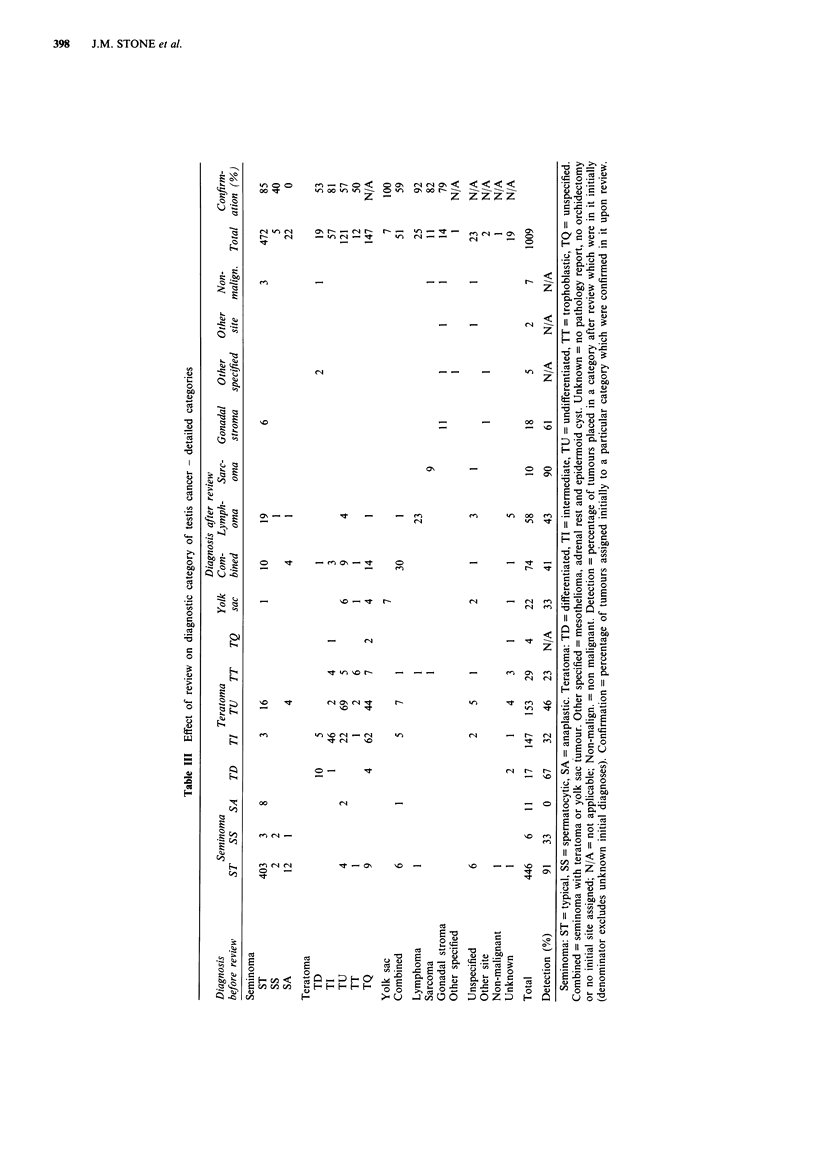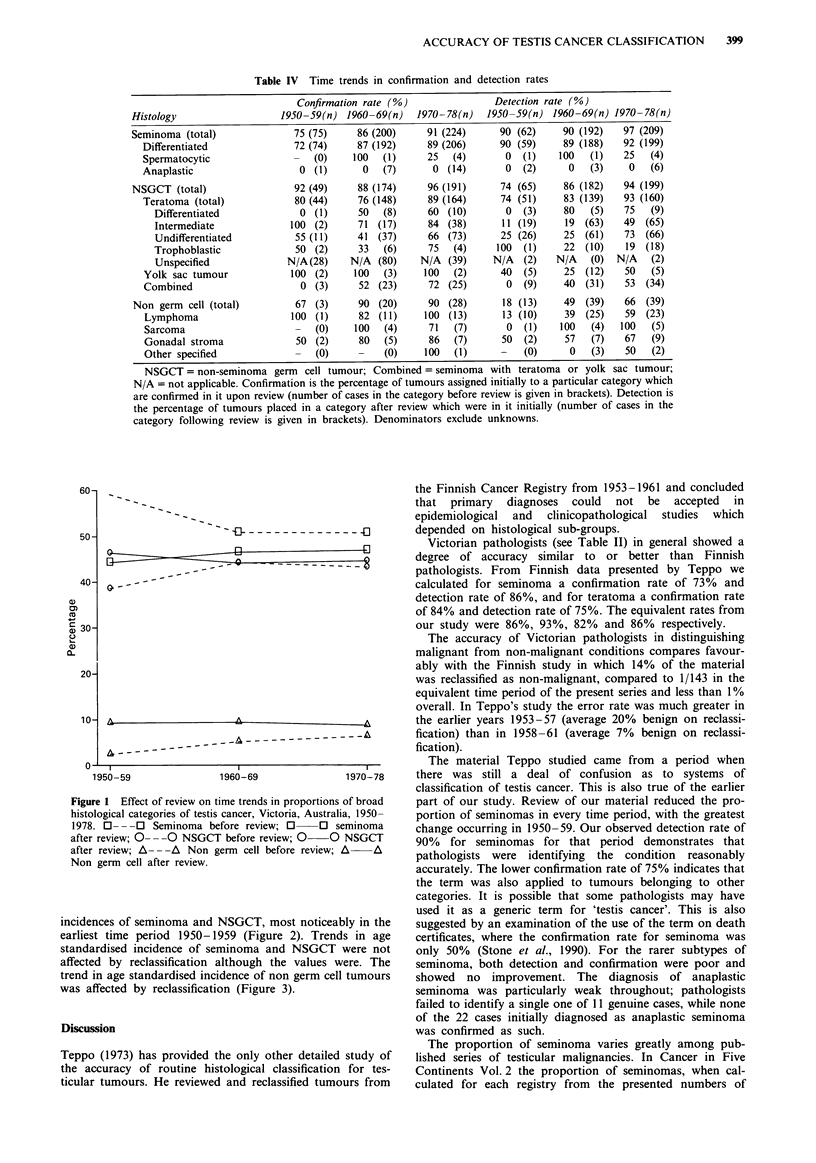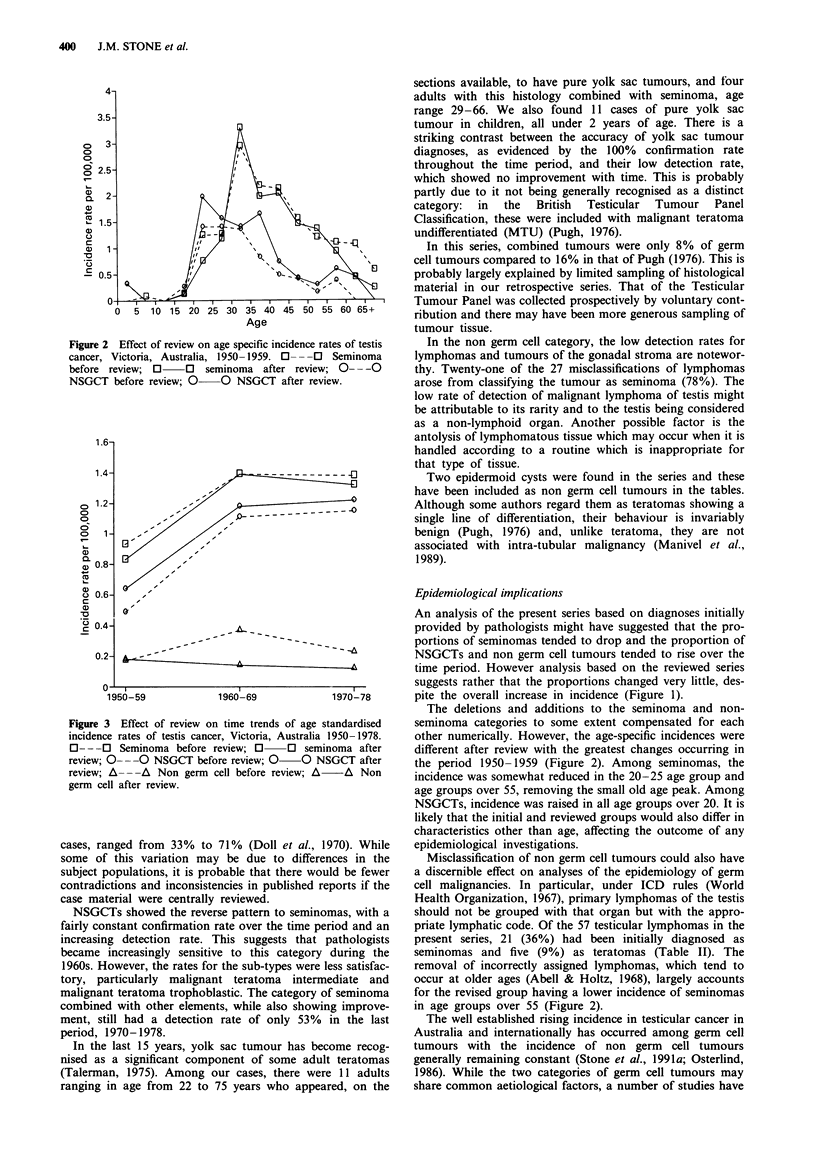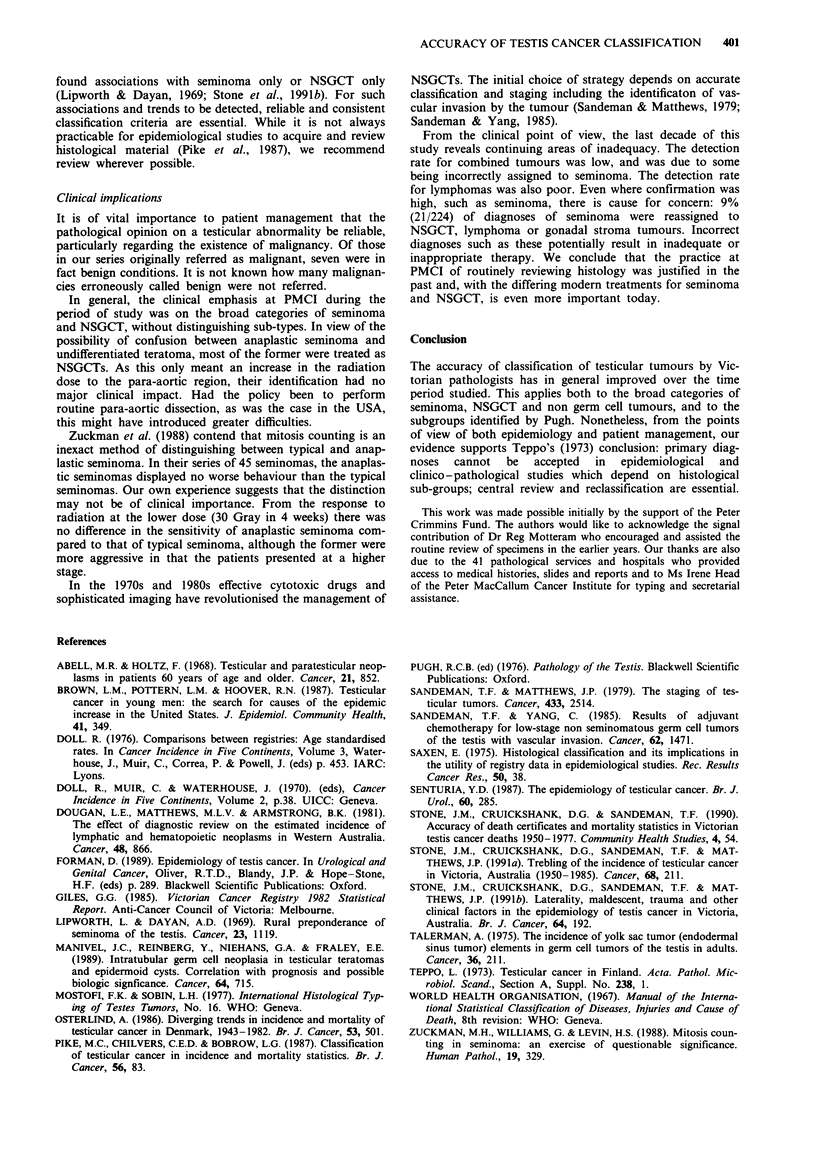# Time trends in accuracy of classification of testicular tumours, with clinical and epidemiological implications.

**DOI:** 10.1038/bjc.1992.276

**Published:** 1992-08

**Authors:** J. M. Stone, T. F. Sandeman, P. Ironside, D. G. Cruickshank, J. P. Matthews

**Affiliations:** Peter MacCallum Cancer Institute, Melbourne, Victoria, Australia.

## Abstract

Initial classifications of 1009 testicular tumours were reviewed as part of a population based survey of all testicular neoplasms in Victoria, Australia, between 1950 and 1978. All reviews were made by one of two pathologists at the Peter MacCallum Cancer Institute, using the system of the British Testicular Tumour Panel. Accuracy of diagnosis varied markedly over the time period and with pathological category. Seven cases were initially designated malignancies but were determined to be non-malignant conditions upon review. In each decade, review reduced the proportion of seminomas and increased the proportion of non-seminoma germ cell tumours (NSGCT) and non germ cell tumours. Reclassification resulted in changed age specific incidences of seminoma and NSGCT, most noticeably in 1950-59. Trends in age standardised incidence of seminoma and NSGCT were not affected by reclassification although the values were. The trend in age standardised incidence of non germ cell tumours was affected by reclassification. The implications of the changes in classification for epidemiological studies and clinical management are discussed.


Br. J. Cancer (1992), 66, 396-401 © Macmillan Press Ltd., 1992

### Time trends in accuracy of classification of testicular tumours, with clinical and epidemiological implications

J.M. Stone, T.F. Sandeman, P. Ironside, D.G. Cruickshank & J.P. Matthews

*Peter MacCallum Cancer Institute, 481 Little Lonsdale Street, Melbourne, Victoria 3000, Australia.*

**Summary** Initial classifications of 1009 testicular tumours were reviewed as part of a population based survey of all testicular neoplasms in Victoria, Australia, between 1950 and 1978. All reviews were made by one of two pathologists at the Peter MacCallum Cancer Institute, using the system of the British Testicular Tumour Panel. Accuracy of diagnosis varied markedly over the time period and with pathological category. Seven cases were initially designated malignancies but were determined to be non-malignant conditions upon review. In each decade, review reduced the proportion of seminomas and increased the proportion of non-seminoma germ cell tumours (NSGCT) and non germ cell tumours. Reclassification resulted in changed age specific incidences of seminoma and NSGCT, most noticeably in 1950-59. Trends in age standardised incidence of seminoma and NSGCT were not affected by reclassification although the values were. The trend in age standardised incidence of non germ cell tumours was affected by reclassification. The implications of the changes in classification for epidemiological studies and clinical management are discussed.

Accurate assignment of a malignancy to the correct pathological category is essential both for treatment of the patient and for scientific investigation of aetiology. In the case of testis cancer, germ cell tumours are distinct in terms of aetiology, behaviour and therapy from other malignancies primary in the testis such as lymphoma, sarcomas and tumours of the gonadal stroma. The major histological breakdown of germ cell tumours is seminoma and non-seminoma (NSGCT). The two categories have distinct age distributions, with NSGCTs concentrated in the age group 15-30 and seminomas about 10 years older (Senturia, 1987). The possibility of different initiating or promoting factors for the two groups makes it desirable that analyses investigating aetiology reliably distinguish between them. Treatment policy also differs markedly between broad groups.

The incidence of germ cell tumours has been increasing internationally (Forman, 1989) and in Australia (Stone *et al.*, 1991*a*) while the rate of occurrence of other testicular malignancies has remained largely unchanged. In the field of testis cancer epidemiology, attention is currently focussed on the identification of aetiological factors underlying the large increase in incidence, which has even been called an epidemic (Brown *et al.*, 1987).

A common source of cases for epidemiological studies is cancer registry data, whose diagnoses are determined by pathologists with varying experience. That diagnoses from such mixed sources are not always reliable or consistent has been shown for thyroid cancer (Saxen *et al.*, 1975) and lymphatic neoplasms (Dougan *et al.*, 1981). In both these studies, diagnostic review of cancer registry material led to substantial reclassifications. Unreliable diagnosis is particularly likely to occur when the tumour is rare and there is no general agreement on classification systems. Such is the case with neoplasms of the testis.

Although some epidemiological studies of testis cancer incorporate review of the pathology of the case material, the only detailed study of the revisions following such review is that of Teppo (1973) who examined testis cancers from the Finnish Cancer Registry. He concluded that primary diagnoses of histological sub groups could not be relied upon.

As part of a population based survey of the epidemiology of testis cancer in the state of Victoria, Australia, we reviewed all available histological material. We present here the results of the review of the pathology of the tumours, the revision in classifications made and briefly discuss the implications for the clinician and the epidemiologist.

### Materials and methods

Compulsory registration of cancer is comparatively recent in Australia. Although the Central Cancer Registry commenced operation in the state of Victoria in 1939 as a hospital based follow-up registry, the statutory collection of all cases of cancer (other than non-melanoma skin cancer) did not start until 1982 (Giles *et al.*, 1985). The Peter MacCallum Cancer Institute (PMCI), a specialist cancer therapy centre, provided virtually all the radiotherapy for the state during the period covered by this study. A substantial proportion of the state's cancer patients are referred to PMCI for treatment, generally after diagnosis at other institutions.

In an attempt to identify all new cases of testis cancer occurring in Victoria between 1950 and 1978, a number of resources were utilised. PMCI medical histories provided the initial data base including private case histories of one of us (TFS) and twelve other radiotherapists associated with PMCI. Since orchidectomy is performed on most testis cancer patients, pathology records offered an effective method of identification of additional cases. A search was made of the records of 27 public hospitals, 12 private pathologists' services, two university pathology departments, the Royal Australian College of Surgeons and the Royal Australian Navy. All pathologists who performed histology for the Victorian population during the period gave access to data, except for one small private service which was no longer operating. In addition, 362 death certificates ascribed to testis cancer for 1950 to 1977 were obtained from the Victorian Registrar of Births, Deaths and Marriages, from a list supplied by the Australian Bureau of Statistics. A detailed account of case ascertainment is given in Stone *et al.* (1991*a*).

In order to investigate the accuracy of the original pathological classifications made, we selected cases diagnosed in the period 1950 to 1978, resident in Victoria, and with histological material available which could be satisfactorily reviewed at PMCI. Sources of cases are presented in Table I. The date of diagnosis was defined as the date of orchidectomy if available, otherwise the date of biopsy of metastasis or post-mortem. Bilateral tumours were treated as a single case, the first malignancy being used for analysis when they were not simultaneous. In one case of simultaneous bilateral germ cell tumours with differing histology, the side with the greater malignancy was used for analysis.

We considered that a single review classification system

Correspondence: J. Stone.
Received 16 October 1991; and in revised form 9 March 1992.

**Table I** Sources of cases of testicular cancer with available pathology Victoria, Australia 1950–1978

| *Source* | *1950–59* | *1960–69* | *1970–78* | *Total* |
|---|---|---|---|---|
| PMCI | 58 | 257 | 174 | 489 |
| TFS | 0 | 6 | 83 | 89 |
| Radiotherapists | 5 | 12 | 11 | 28 |
| Pathologists | 77 | 141 | 180 | 398 |
| Death certificates | 3 | 1 | 1 | 5 |
| Total | 143 | 417 | 449 | 1009 |

PMCI = Peter MacCallum Cancer Institute; TFS = author T.F. Sandeman; Radiotherapists = 12 other radiotherapists in private practice at PMCI; Pathologists = other pathologists or hospitals; Death certificates = cases identified initially through death certificates.

was necessary for the purpose of comparison of specimens from a prolonged time period and a range of pathologists. The pathological classification system of the British Testicular Tumour Panel (Pugh, 1976) was used, with the extension of the category 'pure yolk sac tumour' to include adult as well as juvenile cases. While Pugh's group did not include the category of anaplastic seminoma they did recognise an atypical group of seminoma. We expanded their classification by separating a group corresponding to 'anaplastic seminoma' of Mostofi and Sobin (1977). Tumours stated to be seminoma without specifying a sub-category were grouped with typical seminoma. Interstitial cell tumours and Sertoli cell tumours were grouped together as tumours of the gonadal stroma. Metastases to the testis and non-malignant conditions were included if they were either the initial diagnosis or the review diagnosis but not both.

Some reviews had been done prior to the commencement of this study using alternative classification systems. These diagnostic terms were converted to their equivalents in the British system, with the awareness that the categories do not always completely correspond. This was necessary for 45 (4%) of cases.

All reviews were conducted in the Pathology Department of the PMCI by one of us (PI) or Dr R. Motteram. All cases identified outside PMCI were reviewed during 1978–81 specifically for this study. PMCI usual practice is to review histology of new patients. For 262 (54%) PMCI survey patients it was not considered necessary to conduct another review and that performed at the time of treatment was accepted. The remaining 227 PMCI patients were reviewed at the time of the survey. As a result 184 (38%) of PMCI cases were reviewed twice, with 13 (7%) receiving a major change (seminoma/non-seminoma/non-germ cell) and 29 (15%) a lesser change (sub-groups within a category).

Data analyses were performed on a MicroVax 2 with VMS version 4.7, using purpose written programs. 'Confirmation' is the percentage of tumours assigned initially to a particular category which were confirmed in it upon review. 'Detection' is the percentage of those tumours placed in a category after review which were in it initially. Unknown initial diagnoses were excluded from this calculation.

The 'world standard population' (Doll, 1976) was used in the calculation of age standardised incidences. Calculations of age specific and age standardised incidence were based on all cases which would have been included in an epidemiological analysis and therefore drew on all cases of primary malignant testis cancer in the state of Victoria between 1950 and 1978 identified in the survey, including a small number of cases not histologically verified, and excluding non-malignant conditions and metastases to the testis (Stone *et al.*, 1991*a*). The definition of testis cancer in these calculations was according to the International Classification of Diseases (World Health Organisation, 1967), which excludes lymphomas.

### Results

In total, 1009 eligible cases were identified, 979 (97%) from orchidectomy specimens, 24 (2%) from biopsies of metastases and seven (1%) from post mortems.

The initial and reviewed classifications of the cases are summarised in Table II according to broad categories and in Table III in detail. The 'other specified' category incorporates mesothelioma, adrenal rest and epidermoid cyst. Nineteen cases were assigned an unknown initial diagnosis. For five, no histological examination was performed during life, so reviews were conducted on post-mortem material. The remaining 14 cases were: biopsies from metastases with no primary site determined (10); initial pathology report unavailable (two); orchidectomy done with no classification by the pathologist (one) and orchidectomy done but no information available (one). Seven cases were initially designated malignancies but were determined to be non-malignant conditions upon review: one in 1950–59, four in 1960–69 and two in 1970–78. Four of these displayed chronic inflammation, one trauma, one cystadenoma of the epididymis and the last no tumour at all. At least two of these patients had full courses of radiotherapy. While only limited follow-up information is available, it is known that one died of heart disease at age 78 fourteen years after radiotherapy, and the other, a severely retarded man, died of 'old age' at age 81, thirteen years post-treatment.

Accuracy of diagnosis varied markedly over the time period as shown by the detection and confirmation rates (Table IV). The overall agreement shows a general trend towards improved accuracy. Overall agreement can be defined as the number of tumours placed in the same major histological category (seminoma/NSGCT/non germ cell) both initially and on review, as a percentage of the total number of tumours assigned specified diagnoses of testicular tumour at both classifications. This was calculated as 82% in 1950–59, 88% in 1960–69 and 93% in 1970–78.

In each decade, review reduced the proportion of seminomas in the total series and increased the proportion of NSGCTs and non germ cell tumours (Figure 1). Reclassification of diagnoses resulted in changed age specific

**Table II** Effect of review on diagnostic category of testis cancer – broad categories

| *Diagnosis before review* | *Diagnosis after review* Seminoma | Teratoma | Other germ cell | Non germ cell | Not testis | *Total* | *Confirmation* |
|---|---|---|---|---|---|---|---|
| Seminoma | 431 | 23 | 15 | 27 | 3 | 499 | 86% |
| Teratoma | 16 | 293 | 39 | 7 | 1 | 356 | 82% |
| Other germ cell | 7 | 13 | 37 | 1 | 0 | 58 | 64% |
| Non germ cell | 1 | 2 | 0 | 45 | 3 | 51 | 92% |
| Not testis | 1 | 0 | 0 | 2 | – | 3 | N/A |
| Unknown or unspecified | 7 | 19 | 5 | 9 | 2 | 42 | N/A |
| Total | 463 | 350 | 96 | 91 | 9 | 1009 | |
| Detection | 93% | 86% | 39% | 52% | N/A | | |

Other germ cell = yolk sac tumour, seminoma combined with teratoma or yolk sac tumour. Not testis = metastases to testis, malignancies of other sites and non-malignant conditions. Unknown = no pathology report, no orchidectomy or no initial site assigned. N/A = not applicable. Detection = percentage of tumours placed in a category after review which were in it initially (denominators exclude unknown initial diagnoses). Confirmation = percentage of tumours assigned initially to a particular category which were confirmed in it upon review.

**Table III** Effect of review on diagnostic category of testis cancer – detailed categories

| *Diagnosis before review* | *Seminoma* *ST* | *SS* | *SA* | *Teratoma* *TD* | *TI* | *TU* | *TT* | *TQ* | *Diagnosis after review* *Yolk sac* | *Com-bined* | *Lymph-oma* | *Sarc-oma* | *Gonadal stroma* | *Other specified* | *Other site* | *Non-malign.* | *Total* | *Confirm-ation (%)* |
|---|---|---|---|---|---|---|---|---|---|---|---|---|---|---|---|---|---|---|
| Seminoma | | | | | | | | | | | | | | | | | | |
| ST | 403 | 3 | 8 | | 3 | 16 | | | 1 | 10 | 19 | | 6 | | | 3 | 472 | 85 |
| SS | 2 | 2 | | | | | | | | | 1 | | | | | | 5 | 40 |
| SA | 12 | 1 | | | | 4 | | | | 4 | 1 | | | | | | 22 | 0 |
| Teratoma | | | | | | | | | | | | | | | | | | |
| TD | | | | 10 | 5 | | | | | 1 | | | | 2 | | 1 | 19 | 53 |
| TI | | | | 1 | 46 | 2 | 4 | 1 | | 3 | | | | | | | 57 | 81 |
| TU | 4 | | 2 | | 22 | 69 | 5 | | 6 | 9 | 4 | | | | | | 121 | 57 |
| TT | 1 | | | | 1 | 2 | 6 | | 1 | 1 | | | | | | | 12 | 50 |
| TQ | 9 | | | 4 | 62 | 44 | 7 | 2 | 4 | 14 | 1 | | | | | | 147 | N/A |
| Yolk sac | | | | | | | | | 7 | | | | | | | | 7 | 100 |
| Combined | 6 | | 1 | | 5 | 7 | 1 | | | 30 | 1 | | | | | | 51 | 59 |
| Lymphoma | 1 | | | | | | 1 | | | | 23 | | | | | | 25 | 92 |
| Sarcoma | | | | | | | 1 | | | | | 9 | | | | 1 | 11 | 82 |
| Gonadal stroma | | | | | | | | | | | | | 11 | 1 | 1 | 1 | 14 | 79 |
| Other specified | | | | | | | | | | | | | | 1 | | | 1 | N/A |
| Unspecified | 6 | | | | 2 | 5 | 1 | | 2 | 1 | 3 | 1 | | | 1 | 1 | 23 | N/A |
| Other site | | | | | | | | | | | | | 1 | 1 | | | 2 | N/A |
| Non-malignant | 1 | | | | | | | | | | | | | | | | 1 | N/A |
| Unknown | 1 | | | 2 | 1 | 4 | 3 | 1 | 1 | 1 | 5 | | | | | | 19 | N/A |
| Total | 446 | 6 | 11 | 17 | 147 | 153 | 29 | 4 | 22 | 74 | 58 | 10 | 18 | 5 | 2 | 7 | 1009 | |
| Detection (%) | 91 | 33 | 0 | 67 | 32 | 46 | 23 | N/A | 33 | 41 | 43 | 90 | 61 | N/A | N/A | N/A | | |

Seminoma: ST = typical, SS = spermatocytic, SA = anaplastic. Teratoma: TD = differentiated, TI = intermediate, TU = undifferentiated, TT = trophoblastic, TQ = unspecified. Combined = seminoma with teratoma or yolk sac tumour. Other specified = mesothelioma, adrenal rest and epidermoid cyst. Unknown = no pathology report, no orchidectomy or no initial site assigned; N/A = not applicable; Non-malign. = non malignant. Detection = percentage of tumours placed in a category after review which were in it initially (denominator excludes unknown initial diagnoses). Confirmation = percentage of tumours assigned initially to a particular category which were confirmed in it upon review.

**Table IV** Time trends in confirmation and detection rates

| | *Confirmation rate (%)* | | | *Detection rate (%)* | | |
|---|---|---|---|---|---|---|
| *Histology* | *1950–59(n)* | *1960–69(n)* | *1970–78(n)* | *1950–59(n)* | *1960–69(n)* | *1970–78(n)* |
| Seminoma (total) | 75 (75) | 86 (200) | 91 (224) | 90 (62) | 90 (192) | 97 (209) |
| Differentiated | 72 (74) | 87 (192) | 89 (206) | 90 (59) | 89 (188) | 92 (199) |
| Spermatocytic | – (0) | 100 (1) | 25 (4) | 0 (1) | 100 (1) | 25 (4) |
| Anaplastic | 0 (1) | 0 (7) | 0 (14) | 0 (2) | 0 (3) | 0 (6) |
| NSGCT (total) | 92 (49) | 88 (174) | 96 (191) | 74 (65) | 86 (182) | 94 (199) |
| Teratoma (total) | 80 (44) | 76 (148) | 89 (164) | 74 (51) | 83 (139) | 93 (160) |
| Differentiated | 0 (1) | 50 (8) | 60 (10) | 0 (3) | 80 (5) | 75 (9) |
| Intermediate | 100 (2) | 71 (17) | 84 (38) | 11 (19) | 19 (63) | 49 (65) |
| Undifferentiated | 55 (11) | 41 (37) | 66 (73) | 25 (26) | 25 (61) | 73 (66) |
| Trophoblastic | 50 (2) | 33 (6) | 75 (4) | 100 (1) | 22 (10) | 19 (18) |
| Unspecified | N/A (28) | N/A (80) | N/A (39) | N/A (2) | N/A (0) | N/A (2) |
| Yolk sac tumour | 100 (2) | 100 (3) | 100 (2) | 40 (5) | 25 (12) | 50 (5) |
| Combined | 0 (3) | 52 (23) | 72 (25) | 0 (9) | 40 (31) | 53 (34) |
| Non germ cell (total) | 67 (3) | 90 (20) | 90 (28) | 18 (13) | 49 (39) | 66 (39) |
| Lymphoma | 100 (1) | 82 (11) | 100 (13) | 13 (10) | 39 (25) | 59 (23) |
| Sarcoma | – (0) | 100 (4) | 71 (7) | 0 (1) | 100 (4) | 100 (5) |
| Gonadal stroma | 50 (2) | 80 (5) | 86 (7) | 50 (2) | 57 (7) | 67 (9) |
| Other specified | – (0) | – (0) | 100 (1) | – (0) | 0 (3) | 50 (2) |

NSGCT = non-seminoma germ cell tumour; Combined = seminoma with teratoma or yolk sac tumour; N/A = not applicable. Confirmation is the percentage of tumours assigned initially to a particular category which are confirmed in it upon review (number of cases in the category before review is given in brackets). Detection is the percentage of tumours placed in a category after review which were in it initially (number of cases in the category following review is given in brackets). Denominators exclude unknowns.

**Figure 1** Effect of review on time trends in proportions of broad histological categories of testis cancer, Victoria, Australia, 1950–1978. □- - -□ Seminoma before review; □——□ seminoma after review; O- - -O NSGCT before review; O——O NSGCT after review; Δ- - -Δ Non germ cell before review; Δ——Δ Non germ cell after review.

incidences of seminoma and NSGCT, most noticeably in the earliest time period 1950–1959 (Figure 2). Trends in age standardised incidence of seminoma and NSGCT were not affected by reclassification although the values were. The trend in age standardised incidence of non germ cell tumours was affected by reclassification (Figure 3).

### Discussion

Teppo (1973) has provided the only other detailed study of the accuracy of routine histological classification for testicular tumours. He reviewed and reclassified tumours from the Finnish Cancer Registry from 1953–1961 and concluded that primary diagnoses could not be accepted in epidemiological and clinicopathological studies which depended on histological sub-groups.

Victorian pathologists (see Table II) in general showed a degree of accuracy similar to or better than Finnish pathologists. From Finnish data presented by Teppo we calculated for seminoma a confirmation rate of 73% and detection rate of 86%, and for teratoma a confirmation rate of 84% and detection rate of 75%. The equivalent rates from our study were 86%, 93%, 82% and 86% respectively.

The accuracy of Victorian pathologists in distinguishing malignant from non-malignant conditions compares favourably with the Finnish study in which 14% of the material was reclassified as non-malignant, compared to 1/143 in the equivalent time period of the present series and less than 1% overall. In Teppo's study the error rate was much greater in the earlier years 1953–57 (average 20% benign on reclassification) than in 1958–61 (average 7% benign on reclassification).

The material Teppo studied came from a period when there was still a deal of confusion as to systems of classification of testis cancer. This is also true of the earlier part of our study. Review of our material reduced the proportion of seminomas in every time period, with the greatest change occurring in 1950–59. Our observed detection rate of 90% for seminomas for that period demonstrates that pathologists were identifying the condition reasonably accurately. The lower confirmation rate of 75% indicates that the term was also applied to tumours belonging to other categories. It is possible that some pathologists may have used it as a generic term for 'testis cancer'. This is also suggested by an examination of the use of the term on death certificates, where the confirmation rate for seminoma was only 50% (Stone *et al.*, 1990). For the rarer subtypes of seminoma, both detection and confirmation were poor and showed no improvement. The diagnosis of anaplastic seminoma was particularly weak throughout; pathologists failed to identify a single one of 11 genuine cases, while none of the 22 cases initially diagnosed as anaplastic seminoma was confirmed as such.

The proportion of seminoma varies greatly among published series of testicular malignancies. In Cancer in Five Continents Vol. 2 the proportion of seminomas, when calculated for each registry from the presented numbers of

**Figure 2** Effect of review on age specific incidence rates of testis cancer, Victoria, Australia, 1950–1959. □---□ Seminoma before review; □——□ seminoma after review; O---O NSGCT before review; O——O NSGCT after review.

**Figure 3** Effect of review on time trends of age standardised incidence rates of testis cancer, Victoria, Australia 1950–1978. □---□ Seminoma before review; □——□ seminoma after review; O---O NSGCT before review; O——O NSGCT after review; Δ---Δ Non germ cell before review; Δ——Δ Non germ cell after review.

cases, ranged from 33% to 71% (Doll *et al.*, 1970). While some of this variation may be due to differences in the subject populations, it is probable that there would be fewer contradictions and inconsistencies in published reports if the case material were centrally reviewed.

NSGCTs showed the reverse pattern to seminomas, with a fairly constant confirmation rate over the time period and an increasing detection rate. This suggests that pathologists became increasingly sensitive to this category during the 1960s. However, the rates for the sub-types were less satisfactory, particularly malignant teratoma intermediate and malignant teratoma trophoblastic. The category of seminoma combined with other elements, while also showing improvement, still had a detection rate of only 53% in the last period, 1970–1978.

In the last 15 years, yolk sac tumour has become recognised as a significant component of some adult teratomas (Talerman, 1975). Among our cases, there were 11 adults ranging in age from 22 to 75 years who appeared, on the sections available, to have pure yolk sac tumours, and four adults with this histology combined with seminoma, age range 29–66. We also found 11 cases of pure yolk sac tumour in children, all under 2 years of age. There is a striking contrast between the accuracy of yolk sac tumour diagnoses, as evidenced by the 100% confirmation rate throughout the time period, and their low detection rate, which showed no improvement with time. This is probably partly due to it not being generally recognised as a distinct category: in the British Testicular Tumour Panel Classification, these were included with malignant teratoma undifferentiated (MTU) (Pugh, 1976).

In this series, combined tumours were only 8% of germ cell tumours compared to 16% in that of Pugh (1976). This is probably largely explained by limited sampling of histological material in our retrospective series. That of the Testicular Tumour Panel was collected prospectively by voluntary contribution and there may have been more generous sampling of tumour tissue.

In the non germ cell category, the low detection rates for lymphomas and tumours of the gonadal stroma are noteworthy. Twenty-one of the 27 misclassifications of lymphomas arose from classifying the tumour as seminoma (78%). The low rate of detection of malignant lymphoma of testis might be attributable to its rarity and to the testis being considered as a non-lymphoid organ. Another possible factor is the antolysis of lymphomatous tissue which may occur when it is handled according to a routine which is inappropriate for that type of tissue.

Two epidermoid cysts were found in the series and these have been included as non germ cell tumours in the tables. Although some authors regard them as teratomas showing a single line of differentiation, their behaviour is invariably benign (Pugh, 1976) and, unlike teratoma, they are not associated with intra-tubular malignancy (Manivel *et al.*, 1989).

*Epidemiological implications*

An analysis of the present series based on diagnoses initially provided by pathologists might have suggested that the proportions of seminomas tended to drop and the proportion of NSGCTs and non germ cell tumours tended to rise over the time period. However analysis based on the reviewed series suggests rather that the proportions changed very little, despite the overall increase in incidence (Figure 1).

The deletions and additions to the seminoma and non-seminoma categories to some extent compensated for each other numerically. However, the age-specific incidences were different after review with the greatest changes occurring in the period 1950–1959 (Figure 2). Among seminomas, the incidence was somewhat reduced in the 20–25 age group and age groups over 55, removing the small old age peak. Among NSGCTs, incidence was raised in all age groups over 20. It is likely that the initial and reviewed groups would also differ in characteristics other than age, affecting the outcome of any epidemiological investigations.

Misclassification of non germ cell tumours could also have a discernible effect on analyses of the epidemiology of germ cell malignancies. In particular, under ICD rules (World Health Organization, 1967), primary lymphomas of the testis should not be grouped with that organ but with the appropriate lymphatic code. Of the 57 testicular lymphomas in the present series, 21 (36%) had been initially diagnosed as seminomas and five (9%) as teratomas (Table II). The removal of incorrectly assigned lymphomas, which tend to occur at older ages (Abell & Holtz, 1968), largely accounts for the revised group having a lower incidence of seminomas in age groups over 55 (Figure 2).

The well established rising incidence in testicular cancer in Australia and internationally has occurred among germ cell tumours with the incidence of non germ cell tumours generally remaining constant (Stone *et al.*, 1991*a*; Osterlind, 1986). While the two categories of germ cell tumours may share common aetiological factors, a number of studies have found associations with seminoma only or NSGCT only (Lipworth & Dayan, 1969; Stone *et al.*, 1991*b*). For such associations and trends to be detected, reliable and consistent classification criteria are essential. While it is not always practicable for epidemiological studies to acquire and review histological material (Pike *et al.*, 1987), we recommend review wherever possible.

### *Clinical implications*

It is of vital importance to patient management that the pathological opinion on a testicular abnormality be reliable, particularly regarding the existence of malignancy. Of those in our series originally referred as malignant, seven were in fact benign conditions. It is not known how many malignancies erroneously called benign were not referred.

In general, the clinical emphasis at PMCI during the period of study was on the broad categories of seminoma and NSGCT, without distinguishing sub-types. In view of the possibility of confusion between anaplastic seminoma and undifferentiated teratoma, most of the former were treated as NSGCTs. As this only meant an increase in the radiation dose to the para-aortic region, their identification had no major clinical impact. Had the policy been to perform routine para-aortic dissection, as was the case in the USA, this might have introduced greater difficulties.

Zuckman *et al.* (1988) contend that mitosis counting is an inexact method of distinguishing between typical and anaplastic seminoma. In their series of 45 seminomas, the anaplastic seminomas displayed no worse behaviour than the typical seminomas. Our own experience suggests that the distinction may not be of clinical importance. From the response to radiation at the lower dose (30 Gray in 4 weeks) there was no difference in the sensitivity of anaplastic seminoma compared to that of typical seminoma, although the former were more aggressive in that the patients presented at a higher stage.

In the 1970s and 1980s effective cytotoxic drugs and sophisticated imaging have revolutionised the management of NSGCTs. The initial choice of strategy depends on accurate classification and staging including the identificaton of vascular invasion by the tumour (Sandeman & Matthews, 1979; Sandeman & Yang, 1985).

From the clinical point of view, the last decade of this study reveals continuing areas of inadequacy. The detection rate for combined tumours was low, and was due to some being incorrectly assigned to seminoma. The detection rate for lymphomas was also poor. Even where confirmation was high, such as seminoma, there is cause for concern: 9% (21/224) of diagnoses of seminoma were reassigned to NSGCT, lymphoma or gonadal stroma tumours. Incorrect diagnoses such as these potentially result in inadequate or inappropriate therapy. We conclude that the practice at PMCI of routinely reviewing histology was justified in the past and, with the differing modern treatments for seminoma and NSGCT, is even more important today.

### Conclusion

The accuracy of classification of testicular tumours by Victorian pathologists has in general improved over the time period studied. This applies both to the broad categories of seminoma, NSGCT and non germ cell tumours, and to the subgroups identified by Pugh. Nonetheless, from the points of view of both epidemiology and patient management, our evidence supports Teppo's (1973) conclusion: primary diagnoses cannot be accepted in epidemiological and clinico-pathological studies which depend on histological sub-groups; central review and reclassification are essential.

This work was made possible initially by the support of the Peter Crimmins Fund. The authors would like to acknowledge the signal contribution of Dr Reg Motteram who encouraged and assisted the routine review of specimens in the earlier years. Our thanks are also due to the 41 pathological services and hospitals who provided access to medical histories, slides and reports and to Ms Irene Head of the Peter MacCallum Cancer Institute for typing and secretarial assistance.

### References

ABELL, M.R. & HOLTZ, F. (1968). Testicular and paratesticular neoplasms in patients 60 years of age and older. *Cancer*, **21**, 852.

BROWN, L.M., POTTERN, L.M. & HOOVER, R.N. (1987). Testicular cancer in young men: the search for causes of the epidemic increase in the United States. *J. Epidemiol. Community Health*, **41**, 349.

DOLL. R. (1976). Comparisons between registries: Age standardised rates. In *Cancer Incidence in Five Continents*, Volume 3, Waterhouse, J., Muir, C., Correa, P. & Powell, J. (eds) p. 453. IARC: Lyons.

DOLL, R., MUIR, C. & WATERHOUSE, J. (1970). (eds), *Cancer Incidence in Five Continents*, Volume 2, p.38. UICC: Geneva.

DOUGAN, L.E., MATTHEWS, M.L.V. & ARMSTRONG, B.K. (1981). The effect of diagnostic review on the estimated incidence of lymphatic and hematopoietic neoplasms in Western Australia. *Cancer*, **48**, 866.

FORMAN, D. (1989). Epidemiology of testis cancer. In *Urological and Genital Cancer*, Oliver, R.T.D., Blandy, J.P. & Hope-Stone, H.F. (eds) p. 289. Blackwell Scientific Publications: Oxford.

GILES, G.G. (1985). *Victorian Cancer Registry 1982 Statistical Report*. Anti-Cancer Council of Victoria: Melbourne.

LIPWORTH, L. & DAYAN, A.D. (1969). Rural preponderance of seminoma of the testis. *Cancer*, **23**, 1119.

MANIVEL, J.C., REINBERG, Y., NIEHANS, G.A. & FRALEY, E.E. (1989). Intratubular germ cell neoplasia in testicular teratomas and epidermoid cysts. Correlation with prognosis and possible biologic signficance. *Cancer*, **64**, 715.

MOSTOFI, F.K. & SOBIN, L.H. (1977). *International Histological Typing of Testes Tumors*, No. 16. WHO: Geneva.

OSTERLIND, A. (1986). Diverging trends in incidence and mortality of testicular cancer in Denmark, 1943–1982. *Br. J. Cancer*, **53**, 501.

PIKE, M.C., CHILVERS, C.E.D. & BOBROW, L.G. (1987). Classification of testicular cancer in incidence and mortality statistics. *Br. J. Cancer*, **56**, 83.

PUGH, R.C.B. (ed) (1976). *Pathology of the Testis*. Blackwell Scientific Publications: Oxford.

SANDEMAN, T.F. & MATTHEWS, J.P. (1979). The staging of testicular tumors. *Cancer*, **433**, 2514.

SANDEMAN, T.F. & YANG, C. (1985). Results of adjuvant chemotherapy for low-stage non seminomatous germ cell tumors of the testis with vascular invasion. *Cancer*, **62**, 1471.

SAXEN, E. (1975). Histological classification and its implications in the utility of registry data in epidemiological studies. *Rec. Results Cancer Res.*, **50**, 38.

SENTURIA, Y.D. (1987). The epidemiology of testicular cancer. *Br. J. Urol.*, **60**, 285.

STONE, J.M., CRUICKSHANK, D.G. & SANDEMAN, T.F. (1990). Accuracy of death certificates and mortality statistics in Victorian testis cancer deaths 1950–1977. *Community Health Studies*, **4**, 54.

STONE, J.M., CRUICKSHANK, D.G., SANDEMAN, T.F. & MATTHEWS, J.P. (1991*a*). Trebling of the incidence of testicular cancer in Victoria, Australia (1950–1985). *Cancer*, **68**, 211.

STONE, J.M., CRUICKSHANK, D.G., SANDEMAN, T.F. & MATTHEWS, J.P. (1991*b*). Laterality, maldescent, trauma and other clinical factors in the epidemiology of testis cancer in Victoria, Australia. *Br. J. Cancer*, **64**, 192.

TALERMAN, A. (1975). The incidence of yolk sac tumor (endodermal sinus tumor) elements in germ cell tumors of the testis in adults. *Cancer*, **36**, 211.

TEPPO, L. (1973). Testicular cancer in Finland. *Acta. Pathol. Microbiol. Scand.*, Section A, Suppl. No. **238**, 1.

WORLD HEALTH ORGANISATION, (1967). *Manual of the International Statistical Classification of Diseases, Injuries and Cause of Death*, 8th revision: WHO: Geneva.

ZUCKMAN, M.H., WILLIAMS, G. & LEVIN, H.S. (1988). Mitosis counting in seminoma: an exercise of questionable significance. *Human Pathol.*, **19**, 329.